# The relationship between comprehensive geriatric assessment on the pneumonia prognosis of older adults: a cross-sectional study

**DOI:** 10.1186/s12890-024-03089-4

**Published:** 2024-06-10

**Authors:** Dongmei Li, Hongjuan Jiang, Yanhong Sun, Xiangyu Chi, Xuan Zhang, Hongwen Li

**Affiliations:** Department of Geriatric Respiratory Disease, Shandong Provincial Hospital Affiliated to Shandong First Medical University, 324 Jingwu Weiqi Road, Huaiyin District, Jinan, Shandong Province China

**Keywords:** Geriatric syndrome, Community-acquired pneumonia, Comprehensive geriatric assessment, COVID-19

## Abstract

**Background:**

The mortality of pneumonia in older adults surpasses that of other populations, especially with the prevalence of coronavirus disease 2019 (COVID-19). Under the influence of multiple factors, a series of geriatric syndromes brought on by age is one of the main reasons for the poor prognosis of pneumonia. This study attempts to analyze the impact of geriatric syndrome on the prognosis of pneumonia.

**Methods:**

This is a prospective cross-sectional study. Patients over 65 years old with COVID-19 and severe acute respiratory syndrome coronavirus 2 (SARS-CoV-2)-negative community-acquired pneumonia (SN-CAP) were included in the research. General characteristics, laboratory tests, length of stay (LOS), and comprehensive geriatric assessment (CGA) were collected. Multivariate regression analysis to determine the independent predictors of the severity, mortality, and LOS of COVID-19. At the same time, the enrolled subjects were divided into three categories by clustering analysis of 10 CGA indicators, and their clinical characteristics and prognoses were analyzed.

**Results:**

A total of 792 subjects were included in the study, including 204 subjects of SN-CAP (25.8%) and 588 subjects (74.2%) of COVID-19. There was no significant difference between non-severe COVID-19 and SN-CAP regarding mortality, LOS, and CGA (*P* > 0.05), while severe COVID-19 is significantly higher than both (*P* < 0.05). The Barthel Index used to assess the activities of daily living was an independent risk factor for the severity and mortality of COVID-19 and linearly correlated with the LOS (*P* < 0.05). The cluster analysis based on the CGA indicators divided the geriatric pneumonia patients into three groups: Cluster 1 (*n* = 276), named low ability group, with the worst CGA, laboratory tests, severity, mortality, and LOS; Cluster 3 (*n* = 228), called high ability group with the best above indicators; Cluster 2 (*n* = 288), named medium ability group, falls between the two.

**Conclusion:**

The Barthel Index indicates that decreased activities of daily living are an independent risk factor for the severity, mortality, and LOS of geriatric COVID-19. Geriatric syndrome can help judge the prognosis of pneumonia in older adults.

## Introduction

The coronavirus disease 2019 (COVID-19) is an emergency that overwhelms the global medical capacity, particularly impacting the older generation, and has become the leading cause of geriatric pneumonia death in recent years [1, 2]. For older adults, especially those over 80 years old, it is far from enough to judge the prognosis of pneumonia simply from the age and comorbidities. The lessons learned from aging medicine suggest that patients’ biological age (rather than their actual age) may be critical in systematically assessing infection to avoid excess mortality [1, 3]. Studies have indicated that physiological age is not an independent risk factor for pneumonia in older adults. Instead, influenced by multiple factors, a range of aging-associated geriatric syndromes contribute significantly to the unfavorable prognosis [1, 4–6].

Geriatric syndrome encompasses a cluster of age-related diseases, including cognitive impairment, delirium, falls, frailty, dizziness, syncope, and urinary incontinence [7]. Geriatric syndromes are characterized by a functional decline across multiple physiological systems and are prevalent in people of high biological age. Biological age and geriatric syndromes, as closely related multidimensional concepts, may be captured through multidimensional tools that include biomolecular characteristics.

In countries with an aging population or many older people, physicians should emphasize the function of activities of daily living (ADL) as a prognostic assessment of pneumonia, not just mortality [8]. Numerous studies have revealed that the diseases of poor functional status, multiple comorbidities, frailness, dysphagia, and malnutrition are highly prevalent in geriatric pneumonia and are related to adverse outcomes [9–12]. Additionally, older adults also have an elevated risk of cognitive dysfunction [13, 14], depression [15], and venous thrombosis has been fully demonstrated [16]. Early identification of clinical factors leading to adverse outcomes of geriatric pneumonia may help to understand the natural history of the disease and actively develop treatment methods to alleviate these adverse clinical outcomes.

Severe acute respiratory syndrome coronavirus 2 (SARS-CoV-2) infection is highly heterogeneous, ranging from asymptomatic to mild, moderate, or severe. In addition, the infection can undergo different stages and progress in either direction (improvement and recovery versus deterioration and death). Given the heavy burden of COVID-19 disease and death in older adults, understanding age-related prognostic factors is of great importance for older adults. Early identification of older adults with the risk of severe pneumonia is significant for effectively allocating social resources, saving nursing costs, and rationalizing the allocation of medical resources. Only a few studies have analyzed the prognosis of COVID-19 in geriatric syndrome. This study attempts to analyze the key factors affecting the prognosis of pneumonia in older adults, from age, comorbidities, changes in biomolecules, and comprehensive geriatric assessment (CGA). Therefore, in addition to conventional comorbidities and laboratory tests, we further evaluated pneumonia’s functional status and prognosis in older adults from 10 aspects of geriatric syndrome.

## Methods

### Study design

This is a prospective cross-sectional study on multidimensional assessment of geriatric syndrome and its impact on the prognosis of geriatric pneumonia. Patients with COVID-19 and SARS-CoV-2-negative community-acquired pneumonia (SN-CAP) attending the Department of Geriatrics of the Shandong Provincial Hospital Affiliated to Shandong First Medical University from December 1, 2022, to December 30, 2023, were included in this study.

COVID-19 that meets any of the following conditions and cannot be explained by reasons other than SARS-CoV-2 infection is defined as severe COVID-19: (1) Shortness of breath (RR ≥ 30 times/min); (2) In a resting state, when inhaling air, the oxygen saturation is ≤ 94%; (3) Arterial oxygen partial pressure (PaO_2_) / Fraction of inspiration oxygen (FiO_2_) ≤ 300mmHg; (4) The clinical symptoms gradually worsen, and pulmonary imaging shows significant lesion progression within 24–48 h, with a rate of > 50% [17].

The subjects were comprehensively evaluated from the following ten aspects using various scales: venous thrombosis risk assessment, ADL, frailty, nutritional assessment, cognitive function, depression, fall risk, pressure injury, swallowing function, and urinary incontinence.

### Subjects

Inclusion criteria: (1) Age ⩾ 65 years; (2) Patients diagnosed with COVID-19 and SN-CAP through symptoms, polymerase chain reaction tests, and other clinical examinations. Exclusion criteria: the subject refuses to participate in the study.

Written informed consent was obtained from each participant. The Shandong Provincial Hospital Affiliated to Shandong First Medical University ethics committee approved this study (SZRJJ: No. 2021 − 411).

### Data collection

Age, gender, height, weight, comorbidities, and length of stay (LOS) were collected. Routine blood tests, C-reaction protein (CRP), Serum amyloid A (SAA), procalcitonin (PCT), interleukin-6 (IL-6), liver function test, renal function test, blood electrolytes test, D-dimer, troponin I (TnI), pro-B-type natriuretic peptide (pro-BNP) were obtained from patients’ routine hospital laboratory records upon admission to the ward.

### Comprehensive geriatric assessment

The Barthel Index is used to assess the ADL. This ordinal scale includes ten items of mobility and self-care ADL, evaluating the degree of physical assistance required and the time to perform each item. Scores range from 0 to 100. A higher score reflects the more extraordinary ability to function independently (Scores 0–20: “total” dependency, 21–61: “severe” dependency, 62–90: “moderate” dependency, 91–99: “slight” dependency, 100: “full” independency).

The FRAIL scale is used for frailty assessment. This scale includes five items: fatigue, endurance, free activity ability, illness, and weight loss. Each project scores 1 point (present) or 0 points (not present). A score of 0 indicates non-frail, 1–2 indicates a pre-frail, and 3–5 indicates frailty.

The Mini-Nutritional Assessment (MNA) is used for nutritional assessment. It includes four aspects: anthropometry, overall evaluation, dietary questionnaire, and subjective evaluation. A score of ≥ 24 indicates good nutritional status; 17-23.5 indicates a risk of malnutrition; and < 14 indicates actual malnutrition.

The Water Swallow test assesses patients’ oropharyngeal dysphagia (OD). The patient sits upright, drinks 30 milliliters of warm water, and observes the required time and coughing. It is divided into five levels.

The Braden Scale assesses pressure injury risk. The scale mainly includes assessments of perception, moisture level, mobility, movement, nutritional intake, friction, and shear forces. Each item is rated on a scale of 4, with scores ranging from 1 to 4. A score of ≤ 9 indicates an extremely high risk of pressure injury, while a score of 10–12 indicates a high risk, 13–14 indicates a medium risk, 15–18 indicates a low risk, and a score of over 18 indicates no risk.

The Morse Fall Scale (MFS) assesses fall risk. The scale includes six components: fall history, secondary diagnosis, ambulatory aid, intravenous infusion/heparin lock, gait/transferring, and mental status. The total score is 125 points, with higher scores indicating a greater risk of falls in older adults. A score of < 25 is low risk, 25–45 is moderate risk, and > 45 is high risk.

The Mini-Mental State Examination (MMSE) is a screening tool for evaluating older adults’ cognitive and intellectual function for possible decline. The full scale is divided into five cognitive aspects: orientation, memory, attention and calculation, recall, and language. The total score for the results is 30 points. Whether dementia is related to education level, illiteracy ≤ 17, primary school education level ≤ 20, junior high school, and above education level ≤ 24 points, it is cognitive and intellectual decline.

The Geriatric Depression Scale (GDS-15) contains 15 questions designed to understand older adults’ moods and psychological states over the past week. A score of 0–5 indicates no depression, while a score of 6–9 indicates mild depression and a score of 10–12 indicates severe depression.

The International Continence Control Association Incontinence Questionnaire Short Form (ICI-Q-SF) assesses urinary incontinence by evaluating age, frequency of leakage, amount of leakage, and impact of leakage on daily life. A score of ≤ 7 is mild, 7–14 is moderate, and > 14 is severe.

The Caprini score assesses the risk of venous thromboembolism (VTE) in all hospitalized patients, including 40 risk factors such as general condition, body mass index (BMI), and VTE medical history. The risk factors are assigned values based on the impact of different factors on VTE risk, and the score for each risk factor is 1–5 points. Low risk: 1 point; Medium risk: 2 points; High risk: 3–4 points; Extremely high risk: ≥ 5 points.

### Statistical analysis

The normal distribution data was presented by mean ± standard deviation (SD), compared using the ANOVA test and compared within the group using the Bonferroni method. Non-normally distributed data were shown as median (interquartile range, IQR), and the Kruskal-Wallis test was used for comparison. The categorical variables were expressed by frequency (composition ratio or percentage) and compared by the chi-square test.

Logistics regression analysis was performed on these CGA indicators to determine the independent predictors of the severity and mortality of COVID-19. Receiver operating characteristic (ROC) curves were constructed, and the area under the curves (AUCs) was determined to detect the severity and mortality of COVID-19. The multivariate linear and Cox regression analyses were performed on these CGA indicators simultaneously to determine the independent predictors of the LOS of COVID-19. The Kaplan-Meier method was used to plot survival curves.

We attempted to cluster subjects using the K-means clustering algorithm, a non-hierarchical clustering method [18]. First, we identified three separate cluster centers using cross-validation [19]. The log means, and SD of the above ten CGA indicators was selected as variables to calculate each subject’s Euclidean distance from the cluster’s center, fall into categories according to the principle of the nearest distance, and calculate various categories of new clustering centers. The procedure described above was continuously iterative until the cluster’s center did not change in occurrence or reached our prescribed maximum number of iterations (100). Missing values were imputed to an unknown category. In short, the cluster analysis demonstrated that our participants naturally sorted into three separate clusters (Cluster 1, 2, 3) based on the CGA. These clusters are further discussed below.

A *P* value < 0.05 (two-tailed) was considered statistically significant. Statistical analysis was performed using SPSS version 28.0 (IBM-SPSS, Armonk, NY, USA).

## Result

### General characteristics

A total of 792 subjects were included in the study, including 204 subjects of SN-CAP (25.8%) and 588 subjects (74.2%) of COVID-19. The average age of SN-CAP was 83.4 ± 9.0 years old, and that of COVID-19 was 83.8 ± 7.8 years old. In COVID-19 groups, 73.5% (*n* = 432) of older adults were over 80. Although the average age of severe COVID-19 was slightly higher than that of non-severe COVID-19 and SN-CAP, and BMI was slightly lower than that of SN-CAP, there was no significant statistical difference between the age, sex, and BMI of each group (*P* > 0.05) (Table 1).

**Table 1:** General characteristics of subjects

	SN-CAP *n* = 204	Non-severe COVID-19 *n* = 420	Severe COVID-19 *n* = 168	*P*
Age (year, mean ± SD)	83.4 ± 9.0	83.0 ± 8.0	86.0 ± 7.0	0.230
Sex (man, %)	102 (50.0)	300 (71.4)	96 (57.1)	0.082
BMI (kg/m^2^, mean ± SD)	26.0 ± 5.6	24.3 ± 3.1	23.9 ± 6.9	0.735
Mortality (n, %)	36 (17.6)	6 (1.4)	96 (57.1)	< 0.001^a^
LOS (day, mean ± SD)	13.2 ± 6.6	15.6 ± 11.3	24.4 ± 18.0	0.001^b^

SN-CAP: Severe acute respiratory syndrome coronavirus 2 Negative Community-Acquired Pneumonia, COVID-19: Coronavirus Disease 2019, BMI: Body Mass Index, LOS: length of stay. ^a^ The mortality of severe COVID-19 was significantly higher than that of SN-CAP and non-severe COVID-19 (*P* < 0.001). ^b^ The LOS of severe COVID-19 was significantly higher than that of SN-CAP and non-severe COVID-19 (*P* < 0.001).

Among the patients with COVID-19 included in this study, 420 subjects (71.4%) were non-severe COVID-19, and 168 subjects (28.6%) were severe COVID-19. In terms of the LOS, there was no significant difference between non-severe COVID-19 and SN-CAP (15.6 ± 11.3 vs. 13.2 ± 6.6, *P* = 0.373), while the average LOS of severe COVID-19 was 24.4 ± 18.0 days, which was significantly higher than the two groups (*P* < 0.001). A total of 69 patients (17.4%) died among the subjects included in the analysis. The mortality rate of severe COVID-19 (57.1%) was significantly higher than that of non-severe COVID-19 (1.4%) and SN-CAP (17.6%) (*P* < 0.001).

### Comorbidities

There was an average of 2.4 ± 1.2 comorbidities for SN-CAP, 2.4 ± 1.5 comorbidities for non-severe COVID-19, and 2.5 ± 1.3 comorbidities for severe COVID-19. There was no significant difference in the number of comorbidities among the three groups (*P* = 0.950).

Our study found that 25% of severe COVID-19 subjects were complicated by chronic lung disease, significantly higher than non-severe COVID-19 (8.6%, *P* = 0.047). There was no significant statistical difference between COVID-19 and SN-CAP regarding chronic diseases such as diabetes, hypertension, coronary heart disease, arrhythmia, nervous system diseases, tumors, etc. (*P* > 0.05) (Table 2).

**Table 2:** Comparison of comorbidities in Subjects

Comorbidities (*n*, %)	SN-CAP	Non-severe COVID-19	Severe COVID-19	*P*
Diabetes	66 (32.4)	120 (28.6)	42 (25.0)	0.815
Hypertension	114 (55.9)	234 (55.7)	96 (57.1)	0.991
Coronary heart disease	108 (52.9)	216 (51.4)	72 (42.9)	0.689
Arrhythmias	24 (11.8)	60 (14.3)	12 (7.1)	0.592
Nervous system disease	78 (38.2)	168 (40.0)	96 (57.1)	0.386
Cerebrovascular disease	60 (29.4)	156 (37.1)	78 (46.4)	0.242
Parkinson	12 (5.9)	18 (4.3)	24 (14.3)	0.251
Alzheimer’s	12 (5.9)	12 (2.9)	6 (3.6)	0.765
Chronic pulmonary disease	48 (23.5)	36 (8.6)	42 (25.0)	0.047
COPD	30 (14.7)	24 (5.7)	18 (10.7)	0.317
Asthma	6 (2.9)	6 (1.4)	6 (3.6)	0.778
Interstitial lung disease	12 (5.9)	0 (0)	12 (7.1)	0.044
Obsolete Pulmonary Tuberculosis	0 (0)	18 (4.3)	6 (3.6)	0.293
Pressure sore	6 (2.9)	24 (2.9)	6 (3.6)	0.983
Tumor	36 (17.6)	78 (18.6)	36 (21.4)	0.925
lung cancer	24 (11.8)	24 (5.7)	24 (14.3)	0.339
Other tumors	18 (8.8)	66 (15.7)	18 (7.1)	0.384

SN-CAP: Severe acute respiratory syndrome coronavirus 2 Negative Community-Acquired Pneumonia, COVID-19: Coronavirus Disease 2019, COPD: Chronic Obstructive Pulmonary Disease.

### Laboratory test

Regarding infection-related inflammatory indicators, WBC and IL-6 levels of severe COVID-19 were significantly higher than non-severe COVID-19 (*P* < 0.05). The CRP, SAA, and PCT of severe COVID-19 were considerably more elevated than non-severe COVID-19 and SN-CAP (*P* < 0.05). However, there was no significant difference between non-severe COVID-19 and SN-CAP in various inflammatory indicators (*P* > 0.05) (Table 3).

**Table 3:** Laboratory tests of subjects

	SN-CAP	Non-severe COVID-19	Severe COVID-19	*P* ^a^	*P* ^b^	*P* ^c^
WBC (10^9^/L, mean ± SD)	8.12 ± 2.93	6.16 ± 3.43	8.50 ± 5.07	0.006		0.019
HGB (g/L, mean ± SD)	113 ± 17	119 ± 20	110 ± 23	0.130		
PLT (10^9^/L, mean ± SD)	227 ± 95	196 ± 89	211 ± 88	0.247		
CRP [mg/ml, median (IQR)]	11.62 (5.02, 34.95)	12.59 (4.74, 42.10)	75.01 (45.57, 128.26)	< 0.001	< 0.001	< 0.001
SAA [mg/ml, median (IQR)]	21.92 (4.31, 98.35)	51.13 (6.84, 226.15)	404.50 (101.56, 660.68)	< 0.001	< 0.001	0.001
PCT [ng/ml, median (IQR)]	0.07 (0.05, 0.13)	0.06 (0.03, 0.14)	0.18 (0.08, 0.69)	< 0.001	0.003	0.001
IL-6 [pg/ml, median (IQR)]	16.52 (7.16, 39.43)	8.11 (2.12, 21.60)	31.45 (8.71, 103.78)	0.003		0.004
TnI [ng/ml, median (IQR)]	11.90 (5.63, 25.80)	13.85 (7.49, 19.13)	30.80 (18.28, 40.83)	< 0.001	< 0.001	0.007
Pro-BNP [ng/l, median (IQR)]	363 (149, 1909)	603 (222, 1517)	1010 (450, 3052)	0.087		
AST [U/L, median (IQR)]	21 (17, 31)	22 (16, 35)	37 (28, 52)	< 0.001	0.002	0.001
ALT [U/L, median (IQR)]	13 (10, 20)	17 (11, 30)	24 (16, 30)	0.125		
GGT [U/L, median (IQR)]	35 (21, 55)	32 (18, 51)	33.5 (22, 46)	0.989		
ALB (g/l, mean ± SD)	34.1 ± 5.4	33.8 ± 4.5	31.7 ± 5.1	0.114		
CR [µmol/l, median (IQR) ]	65.7 (57.1, 79.7)	71.7 (57.7, 86.3)	77.5 (60.2, 100.6)	0.190		
K^+^ (mmol/l, mean ± SD)	3.93 ± 0.44	3.91 ± 0.49	4.06 ± 0.69	0.433		
Na^+^ (mmol/l, mean ± SD)	135.6 ± 6.4	136.4 ± 4.6	136.6 ± 7.7	0.795		
D-Dimer [mg/l, median (IQR)]	1.02 (0.70, 1.37)	0.82 (0.45, 1.35)	1.45 (0.75, 3.11)	0.006		0.005

SN-CAP: Severe acute respiratory syndrome coronavirus 2 Negative Community-Acquired Pneumonia, COVID-19: Coronavirus Disease 2019, WBC: white blood cell, HGB: hemoglobin, PLT: platelet, CRP: C-reaction protein, SAA: Serum amyloid A, PCT: procalcitonin, IL-6: interleukin-6, TnI: troponin I, pro-BNP: pro-brain natriuretic peptide, AST: aspartate aminotransferase, ALT: alanine aminotransferase, GGT: γ-glutamyl transferase, ALB: albumin, CR: creatinine. ^a^ Comparison between three groups, ^b^ Severe COVID-19 vs. SN-CAP, ^c^ Severe COVID-19 vs. Non-severe COVID-19.

Regarding other laboratory tests, the TnI and AST of severe COVID-19 were significantly higher than non-severe COVID-19 and SN-CAP (*P* < 0.05). The D-Dimer of severe COVID-19 patients was considerably higher than non-severe COVID-19 patients (*P* < 0.05) (Table 3).

### Comprehensive geriatric assessment

In our research, we found that 85.7% of the patients with severe COVID-19 were completely/seriously unable to take care of themselves, and 89.3% of them had a frailty state. We also found that whether it was non-severe COVID-19 or severe COVID-19, both had varying degrees of malnutrition. As we can see, 46.4% of severe COVID-19 had dysphagia, and 32.1% of them had a high/extreme risk of pressure injury, which was significantly higher than non-severe COVID-19 (10% separately, *P* < 0.05). While 92.9% of severe COVID-19 had an increased risk of falling, and 57.1% of them had moderate/severe urinary incontinence. Our study found that 96.4% of severe COVID-19 had different degrees of cognitive and intellectual decline. GDS15 assessed that 46.4% of severe COVID-19 had various degrees of depression, while only 5.7% of non-severe COVID-19 had depression (*P* < 0.05). The Caprini score showed that 92.9% of severe COVID-19 VTE risk is extremely high, and the proportion of non-severe COVID-19 is as high as 50%.

From Table 4, we find that in all indicators of the CGA, severe COVID-19 was significantly worse than SN-CAP and non-severe COVID-19 (*P* < 0.05). However, there was no significant difference between SN-CAP and non-severe COVID-19 (*P* > 0.05).

**Table 4:** Comprehensive geriatric assessment of subjects

	SN-CAP	Non-severe COVID-19	Severe COVID-19	*P* ^a^	*P* ^b^	*P* ^c^
Barthel index	62.1 ± 33.4	61.9 ± 29.0	28.9 ± 24.4	< 0.001	< 0.001	< 0.001
Frail scale	2.7 ± 1.5	2.6 ± 1.3	3.6 ± 0.9	0.002	0.006	< 0.001
MNA	10.7 ± 1.7	10.7 ± 2.6	8.5 ± 2.2	< 0.001	0.001	< 0.001
water swallow test	1.5 ± 0.7	1.8 ± 1.2	2.8 ± 1.4	< 0.001	< 0.001	< 0.001
Braden scale	18.8 ± 4.5	18.9 ± 6.3	14.6 ± 4.0	0.002	0.005	0.001
Morse fall scale	49.3 ± 12.2	54.7 ± 14.3	60.2 ± 9.8	0.009	0.002	0.059
MMSE	21.2 ± 8.6	19.2 ± 8.6	12.0 ± 7.7	< 0.001	< 0.001	< 0.001
GDS-15	2 (0, 4)	2 (0, 4)	5 (1, 8)	0.017	0.029	0.034
ICI-Q-SF	0 (0, 8)	0 (0, 6)	9 (4, 18)	< 0.001	0.005	< 0.001
Caprini score	4.5 ± 1.1	4.8 ± 1.7	5.9 ± 1.5	0.001	0.001	0.001

SN-CAP: Severe acute respiratory syndrome coronavirus 2 Negative Community-Acquired Pneumonia, COVID-19: Coronavirus Disease 2019, Barthel index: activity of daily living scale, MNA: mini-nutritional assessment, Braden scale: pressure injury scale, MMSE: Mini-mental State Examination, GDS-15: Geriatric Depression Scale 15, ICI-Q-SF: International Continence Control Association Incontinence Questionnaire Short Form, Caprini score: venous thromboembolism risk assessment. ^a^ Comparison between three groups, ^b^ Severe COVID-19 vs. SN-CAP, ^c^ Severe COVID-19 vs. Non-severe COVID-19.

### The impact of geriatric syndromes on mortality, length of stay, and severity of COVID-19

**Fig. 1:**
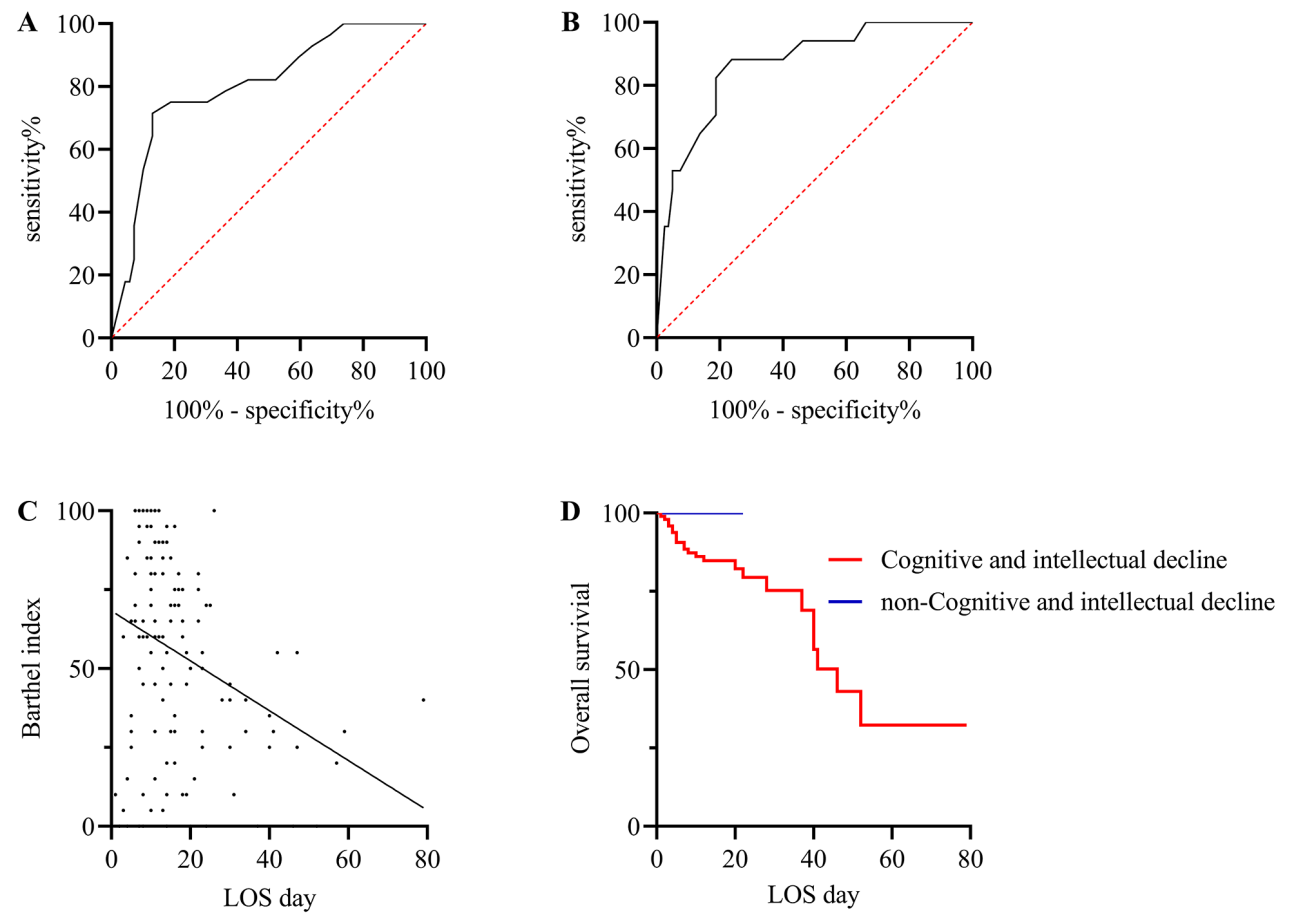
Predictors for discriminating the prognosis of COVID-19 using multiple regression analysis. **(A)** Receiver operating characteristic (ROC) curves for the Barthel Index to detect the severity of COVID-19. **(B)** ROC curves for the Barthel Index to detect the mortality of COVID-19. **(C)** The Barthel Index was linearly correlated with LOS. **(D)** The survival curves for the Mini-Mental State Examination (MMSE) of COVID-19. LOS: length of stay

After excluding the effects of inflammation indicators, LOS, pulmonary comorbidities, and mortality, logistic regression analysis showed that the Barthel Index was an independent risk factor for the severity of geriatric COVID-19 (*P* = 0.003). According to the ROC curve, Barthel Index < 37.5 was the best marker to detect the risk of severe COVID-19, with a sensitivity of 71.43% and specificity of 86.96% (AUC 0.803, *95% CI* 0.706–0.901; *P* < 0.001) (Fig. 1A). At the same time, after excluding the effects of inflammation indicators, LOS, pulmonary comorbidities, and severity, the Barthel Index is also an independent risk factor for the mortality of geriatric COVID-19 (*P* < 0.001). The ROC curve showed that Barthel Index < 42.5 was a marker to judge the risk of death from COVID-19, with a sensitivity of 88.24% and specificity of 76.25% (AUC 0.868, *95% CI* 0.777–0.958; *P* < 0.0001) (Fig. 1B).

After multiple linear regression analyses, excluding the effects of inflammation indicators, pulmonary comorbidities, and severity of COVID-19, the Barthel Index was linearly correlated with LOS (*P* = 0.002) (Fig. 1C). In the COX regression analysis, MMSE was an independent predictor of LOS after excluding the influencing factors mentioned above (*P* < 0.001). From Fig. 1D, we can see that the LOS and mortality of COVID-19 with cognitive and intellectual decline were significantly higher than those of COVID-19 without cognitive and intellectual decline (*P* < 0.001).

### Cluster analysis of geriatric pneumonia

**Fig. 2:**
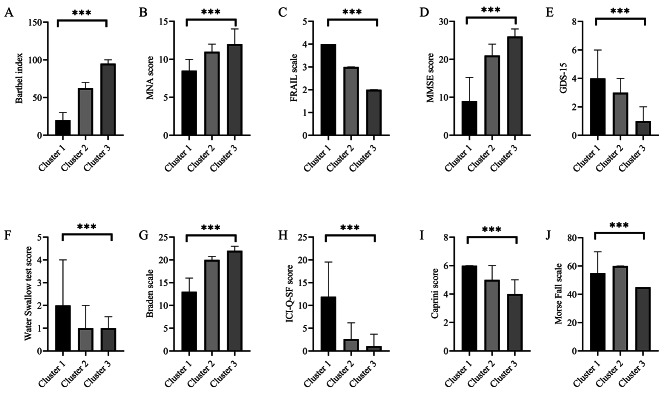
The comprehensive geriatric assessment indicators of the three Clusters. **(A)** Barthel Index **(B)** MNA **(C)** Braden Scale **(D)** MMSE **(E)** Morse Fall Scale **(F)** FRAIL scale **(G)** Water Swallow test **(H)** GDS-15 **(I)** ICI-Q-SF **(J)** Caprini score. MNA: Mini-Nutritional Assessment, MMSE: Mini-Mental State Examination, GDS-15: Geriatric Depression Scale, ICI-Q-SF: International Continence Control Association Incontinence Questionnaire Short Form Columns and error bars represented the median (IQR). ^***^p < 0.001

We conducted cluster analysis on 10 CGA indicators, dividing the geriatric pneumonia patients into three groups and naming them as Cluster 1 low ability group (*n* = 276), Cluster 2 medium ability group (*n* = 288), and Cluster 3 high ability group (*n* = 228). From Fig. 2, we can see that Cluster 1 has the worst self-care ability (Fig. 2A), nutritional status (Fig. 2B), frailty (Fig. 2C), cognitive and intellectual status (Fig. 2D), depression (Fig. 2E), and swallowing function (Fig. 2F) among the three groups. At the same time, the risk of pressure ulcers (Fig. 2G), urinary incontinence (Fig. 2H), and VTE (Fig. 2I) in Cluster 1 was the highest among the three groups. Although there was no significant difference in the risk of falls between Cluster 2 and Cluster 1 (*P* = 0.744, Fig. 2J), all other CGA aspects in Cluster 2 were significantly better than Cluster 1 (*P* < 0.05). Cluster 3 was a high-ability group, and except for no significant difference in swallowing function compared to Cluster 2 (*P* = 0.385, Fig. 2F), its CGA indicators are significantly better than the other two groups (*P* < 0.05).

**Fig. 3:**
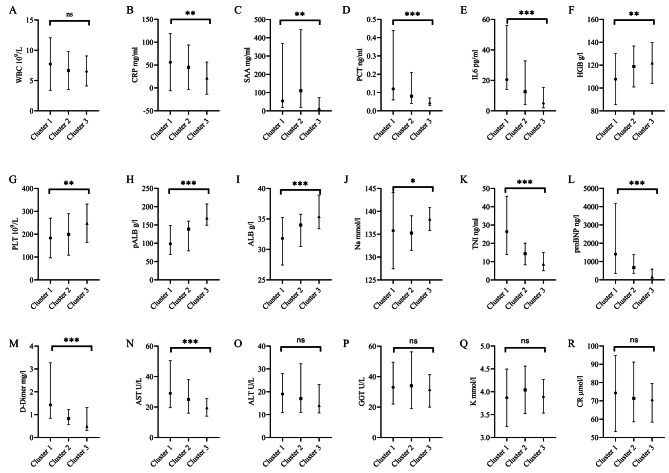
The laboratory tests of the three Clusters. **(A)** WBC **(B)** HGB **(C)** PLT **(D)** CRP **(E)** SAA **(F)** PCT **(G)** IL6 (H) TNI **(I)** proBNP **(J)** D-Dimer **(K)** K^+^**(L)** Na^+^**(M)** AST **(N)** ALT **(O)** GGT **(P)** pALB **(Q)** ALB **(R)** CR. WBC: white blood cell, HGB: hemoglobin, PLT: platelet, CRP: C-reaction protein, SAA: Serum amyloid A, PCT: procalcitonin, IL6: interleukin 6, TnI: troponin I, pro-BNP: pro-brain natriuretic peptide, AST: aspartate aminotransferase, ALT: alanine aminotransferase, GGT: γ-glutamyl transferase, ALB: albumin, CR: creatinine. Columns and error bars represented the median (IQR). ^*^p < 0.05, ^**^p < 0.01, ^***^p < 0.001, ns: no significant

For the laboratory testing, in terms of inflammatory indicators, the WBC among the three groups were not significantly different (*P* > 0.05, Fig. 3A), while the CRP, SAA, PCT, and IL-6 of Cluster 3 were considerably lower than those of Cluster 1 and Cluster 2 (*P* < 0.05, Fig. 3B-E). The levels of hemoglobin (HGB), platelet (PLT), pro-albumin (pALB), ALB, and Na in the Cluster 3 group were significantly higher than those in Cluster 1 (*P* < 0.05, Fig. 3F-J). In comparison, the levels of TNI, proBNP, D-Dimer, and aspartate aminotransferase (AST) were significantly lower than those in Cluster 1 (*P* < 0.05, Fig. 3K-N). Other laboratory indicators had no significant difference (Fig. 3O-R).

**Fig. 4:**
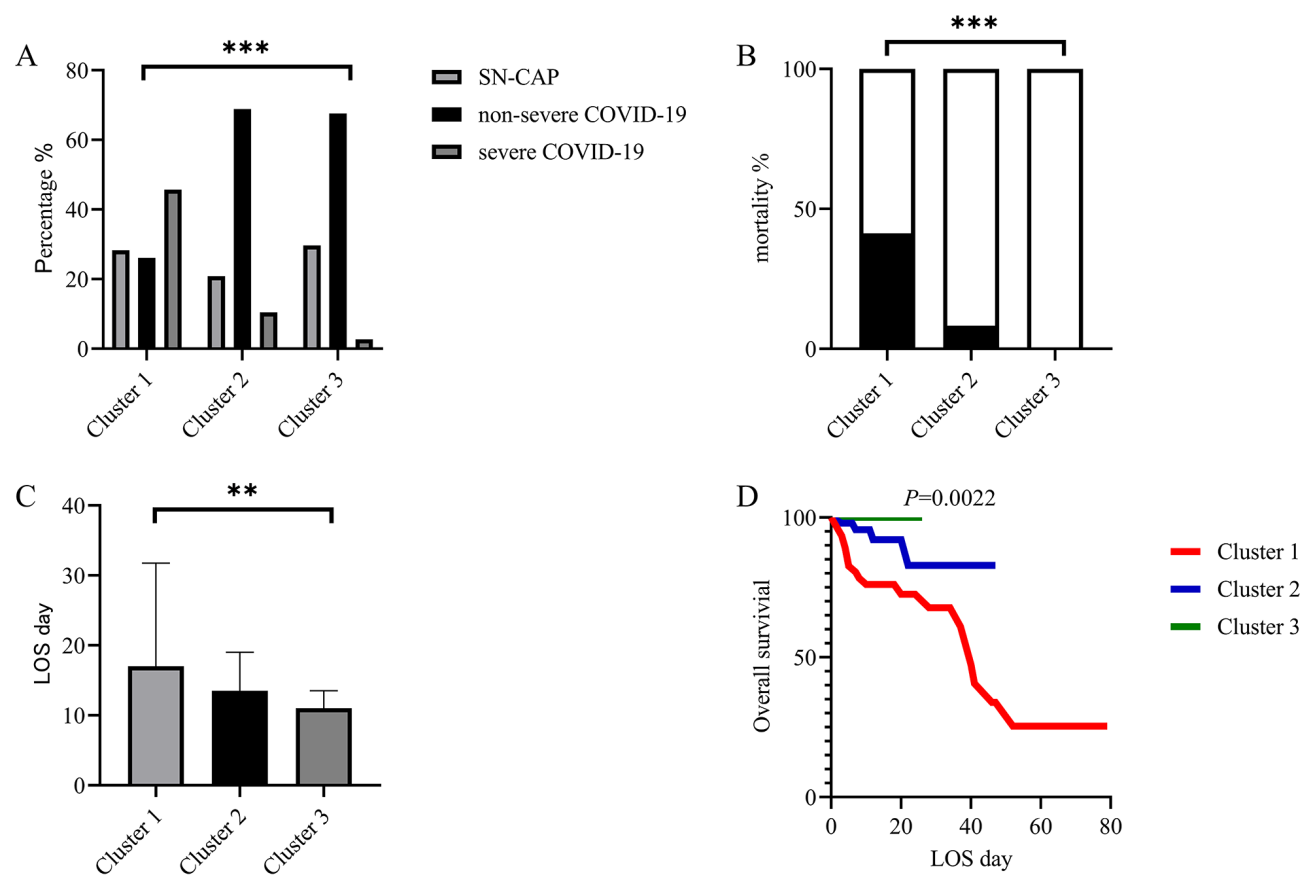
The prognosis of the three Clusters. **(A)** The severity classification of the three Clusters. **(B)** The mortality of the three Clusters. **(C)** The LOS of the three Clusters. **(D)** The survival curves for the three Clusters. LOS: length of stay. ^**^p < 0.01, ^***^p < 0.001

As for prognosis, the proportion of severe COVID-19 in Cluster 3 (Fig. 4A), mortality (Fig. 4B), and LOS (Fig. 4C) were significantly lower than those in Cluster 1. From Fig. 4D, we can more intuitively observe that Cluster 3 has the shortest LOS and the lowest mortality. Cluster 1 has the worst prognosis among the three groups, while Cluster 2 is in the middle.

## Discussion

In a comprehensive assessment of older patients with pneumonia, our study revealed that patients with severe COVID-19 exhibited higher levels of inflammatory markers and poorer GCA scores and prognosis than those with SN-CAP and non-severe COVID-19. We identified the Barthel Index as an independent predictor of COVID-19 progression. Concurrently, we performed a cluster analysis and stratified patients into three categories based on their CGA scores to enhance the prognostic evaluation of pneumonia in older adults.

During the COVID-19 pandemic, it was found that the disorder of ADL at admission is associated with a greater risk of functional decline [20]. Many studies suggest also that frailty is related to the hospitalization rate, severity, mortality rate, and LOS [5, 21–23]. The higher incidence of malnutrition in hospitalized patients is an ominous sign of poor prognosis [24–27]. In addition, the invasion of the SARS-CoV-2 into peripheral nerves can lead to loss of smell, impaired sensory function of the oropharynx, swallowing disorders, and an increased risk of death [28]. Although these geriatric syndromes are different, there is often a correlation and overlap among poor ADL, frailty, malnutrition, and dysphagia in older adults [29, 30]. Interestingly, these conditions have inflammatory characteristics and acute or chronic nutritional deficiencies, which may be related to immune aging caused by immune impairment in older adults, inflammation, and physiological vulnerability to endogenous or exogenous stressors such as drugs or infections [31, 32]. In addition, the risk of falls and urinary incontinence increased significantly due to the deterioration of physical functions [33, 34]. Factors such as inactivity, decreased tissue perfusion, excessive inflammation, and the use of vasopressors, as well as microvascular thrombosis, significantly increase the risk of pressure ulcers [35]. These factors put forward higher requirements for the family and medical care of older patients with pneumonia [34].

At the same time, our study also analyzed the independent risk factors for the prognosis of COVID-19 in geriatric syndrome. The Barthel Index was an independent risk factor for the severity and mortality. This is consistent with previous research results [36–39]. At the same time, the Barthel Index was independently and linearly correlated with the LOS, with poorer ADL resulting in more extended hospital stays. The COX regression analysis found that MMSE was an independent factor in predicting adverse outcomes [40]. The COVID-19 pandemic has been a long-term stressor, which significantly affects the health-related quality of life and may lead to greater cognitive and functional vulnerability in patients [41]. Therefore, specific prevention and intervention plans to repair cognitive deficits will be essential to achieving independent functioning and improving the quality of life for many patients [40].

Physical disabilities and impaired daily living activities can reduce the quality of life and functional independence of patients, thereby increasing the necessity of long-term care services. Given these pieces of evidence, it seems clear that early diagnosis and treatment optimization of geriatric syndrome will also reduce the economic impact on healthcare systems and individual socio-economic burdens. The previous studies above show that factors such as daily activity ability, fatigue, swallowing dysfunction, malnutrition, urinary incontinence, and the risk of venous thrombosis are all associated with poor prognosis of geriatric pneumonia. Our findings indicate no significant difference in outcome between non-severe COVID-19 and SN-CAP in older adults. Consequently, healthcare and community workers are advised to focus on older adults exhibiting more pronounced geriatric syndromes.

To fully utilize the comprehensive assessment of the older adults to determine the prognosis of geriatric pneumonia, we conducted cluster analysis based on its CGA level. Cluster1 is a low-ability group, with the worst CGA index among the three groups, indicating that these subjects often have partial or complete inability to take care of themselves, malnutrition, frailty, cognitive and intellectual decline, dysphagia, depression, and the risk of pressure ulcers, urinary incontinence, and VTE is the highest. At the same time, in terms of laboratory testing, Cluster 1 subjects also performed poorly. All these lead to a high proportion of severe COVID-19 in Cluster 1, a prolonged LOS, and a high mortality. Cluster 1 is the group with the worst prognosis for pneumonia in geriatrics. Cluster 3 has the best CGA indicators, and its laboratory tests and prognosis are also the best among the three groups. And Cluster 2 falls between the two.

It is emphasized that the comprehensive assessment of older adults plays a vital role in judging the prognosis of pneumonia for geriatric. Especially for older people in nursing facilities and home care, a multidimensional assessment of pneumonia through a simple scale may help to select a more favorable medical resource allocation scheme for pneumonia patients from the limited social resources (home care or treatment in a first-class hospital or a tertiary hospital or ICU). Research shows that multi-field assessment of hospitalized older patients with pneumonia may also help to plan nursing, rehabilitation, and follow-up after discharge [20]. Although the COVID-19 pandemic has ended, this study still has enlightenment significance for caring for older adults in the epidemic.

This study still has many limitations. Firstly, due to the single-center nature of the study, the sample size is small, and the reliability of the multi-factor analysis is reduced. Secondly, this study is a cross-sectional study, which failed to evaluate the long-term and short-term effects of pneumonia on geriatric syndrome after discharge from the hospital. Thirdly, despite the addition of multiple indicators for evaluation, there is still a lack of assessment of geriatric syndromes such as sarcopenia, which is a progressive systemic musculoskeletal disorder with an increased risk of adverse events such as falls and fractures, mobility disorders, heart and respiratory diseases, cognitive impairment, hospitalization, and death [42]. In addition, this study only used the Barthel index to evaluate simple daily activities. In contrast, instrumental daily living activities were affected in older adults before simple daily activities, but we did not evaluate them.

In conclusion, our research found no significant difference between non-severe COVID-19 and SN-CAP in mortality, LOS, and CGA, while severe COVID-19 was significantly higher than both. The Barthel Index indicated that the decrease in ADL was an independent risk factor for the prognosis of COVID-19. Meanwhile, through clustering analysis of CGA indicators, we classified pneumonia of geriatrics into three clusters (cluster 1 low-ability, cluster 2 moderate-ability, and cluster 3 high-ability). People in Cluster 1 often indicate the worst prognosis of pneumonia. Multidimensional assessment, including CGA, can help judge the prognosis of pneumonia in geriatrics and help select a more favorable medical resource allocation scheme for pneumonia from the limited social resources. Clinicians will benefit from standardized assessments of elderly syndrome in hospitalized patients and incorporate them into clinical guidelines to make comprehensive clinical decisions on treatment methods and early rehabilitation strategies for these diseases. We recommend early, active, and proactive multimodal interventions to reduce the high mortality rate of elderly pneumonia.

## Data Availability

The data presented in this study are available only upon request from the corresponding author.

## Abbreviations

COVID-19: coronavirus disease 2019
SARS-CoV-2: severe acute respiratory syndrome coronavirus 2
CGA: comprehensive geriatric assessment
SN-CAP: SARS-CoV-2-negative community-acquired pneumonia
ADL: activity of daily living
LOS: length of stay
PaO_2_: arterial oxygen partial pressure
FiO_2_: fraction of inspiration oxygen
CRP: C-reaction protein
SAA: Serum amyloid A
PCT: procalcitonin
IL-6: interleukin-6
TnI: troponin I
pro-BNP: pro-B-type natriuretic peptide
MNA: Mini-Nutritional Assessment
OD: oropharyngeal dysphagia
MFS: Morse fall scale
MMSE: Mini-mental State Examination
GDS-15: Geriatric Depression Scale
ICI-Q-SF: International Continence Control Association Incontinence Questionnaire Short Form
VTE: venous thromboembolism
BMI: body mass index
SD: standard deviation
IQR: interquartile range
