# Correction: The relationship between comprehensive geriatric assessment on the pneumonia prognosis of older adults: a cross-sectional study

**DOI:** 10.1186/s12890-024-03143-1

**Published:** 2024-07-04

**Authors:** Dongmei Li, Hongjuan Jiang, Yanhong Sun, Xiangyu Chi, Xuan Zhang, Hongwen Li

**Affiliations:** Department of Geriatric Respiratory Disease, Shandong Provincial Hospital Affiliated to Shandong First Medical University, 324 Jingwu Weiqi Road, Huaiyin District, Jinan, Shandong Province China

**Correction: BMC Pulmonary Medicine (2024) 24:276**

10.1186/s12890-024-03089-4

Following publication of the original article, it came to the authors’ attention that there was an error in the affiliation information: where it should say ‘Shandong Provincial Hospital Affiliated to Shandong First Medical University’, it said ‘Shandong Provincial Hospital, Shandong First Medical University’. The affiliation has since been corrected in the article. The authors thank you for reading and apologize for any inconvenience caused.

